# Cognitive glucose sensitivity—proposing a link between cognitive performance and reliance on external glucose uptake

**DOI:** 10.1038/s41387-022-00191-6

**Published:** 2022-03-14

**Authors:** Tobias Neukirchen, Ralph Radach, Christian Vorstius

**Affiliations:** University of Wuppertal, Department of Psychology, Wuppertal, Germany

**Keywords:** Obesity, Cognitive control, Obesity, Risk factors, Pre-diabetes

## Abstract

Existing evidence on the effects of glucose supplementation on cognitive performance appears inconclusive. Metabolic switching offers an approach to explain such incoherent findings based on differences in cognitive functioning after fasting. We propose a new construct, cognitive glucose sensitivity (CGS), which quantifies individual performance gain due to glucose supplementation. We tested the hypothesis that the effects of glucose ingestion depend on CGS, cognitive task domain, and sex. In addition, the relationship between CGS and body mass index (BMI) was examined. Seventy-one participants (48 female) were tested in two conditions each (deprivation baseline vs. glucose supplementation), performing tasks from different cognitive domains (memory and executive functioning). We found significant evidence for a correlation of deprivation baseline performance and CGS across domains (Corsi-Block-Tapping Task: *r* = −0.57, *p* < 0.001; Go-No-Go Task: *r* = 0.39, *p* *=* 0.001; word list recall: *r* = −0.50, *p* < 0.001). Moreover, individual CGS differed significantly between tasks (*p* = 0.018). Only in men, BMI was significantly related to CGS in a word recall paradigm (*r* = 0.49, *p* = 0.017). Ou*r* findings support the notion that the effects of glucose depend on CGS, task domain, and sex. The effort to reduce performance impairment (short-term) might sacrifice independence from external glucose (long term), possibly via declining blood glucose regulation. Therefore, CGS could be regarded as a candidate to enhance our understanding of the etiology of unhealthy eating.

## Introduction

The cognitive effects of glucose supplementation vary widely between individuals. This is reflected in a controversial discussion in the literature over the past decades. Reported findings in this debate have been contradictory [1–8] with some studies showing that glucose intake improves cognitive performance only under specific circumstances or can even have adverse effects [4–6]. Such findings are in line with the assumption that a healthy human body is able to produce glucose in sufficient amounts by itself, therefore cognitive performance should be independent of external glucose intake. However, a number of studies also provide evidence that (external) glucose supplementation indeed does improve cognitive performance [7, 8].

Taking these findings into account, we suggest a new construct, namely an individual cognitive glucose sensitivity (CGS), which we define as the degree of glucose dependence of cognitive performance. This CGS corresponds to the individual increase in performance as a result of glucose intake, compared with baseline performance (without glucose intake).

Against this background, we hypothesize that a benefit of glucose supplementation on cognitive performance is moderated by individual variables (deprivation baseline performance, weight, sex) and performance domain (e.g., memory vs. executive functions). This idea is consistent with the findings of others [2, 5] and offers the opportunity to integrate recent findings on inter-individual differences in glucose metabolism at the neurobiological level [9]. The present study reflects the first attempt to quantify individual CGS, to lay the foundation for a useful descriptive performance parameter, exposing cognitive performance impairments that might result from weaknesses in metabolic switching [10]. The new construct, therefore, presents a potentially important factor in eating behavior and the development of diabetes, as it could impact individual food intake to prevent negative cognitive consequences that can be caused by hypoglycemia [11].

## Method

### Study design and sample

We implemented a within-subject design with randomized order of glucose supplementation (glucose vs. baseline) to observe inter-individual cognitive performance differences. Each participant was tested on 2 consecutive days, with sessions differing only in terms of supplementation condition and (parallel) test versions of the cognitive paradigms. Testing was scheduled at the same time of day for both sessions after fasting for 12 hours (hydration with water was permitted). Participants were randomly assigned to predetermined, counterbalanced sequences of supplementation conditions and parallel test versions of cognitive tasks. On the day of glucose supplementation, a solution consisting of 200 ml water and 75 g glucose was ingested orally. The dosage was based on WHO recommendations for investigating glucose tolerance [12]. On the baseline day, the same amount of water without glucose was consumed. To minimize confounding effects, participants and conducting research assistants were blind to which substance was consumed in each session (and were told only that one beverage was sweetened). Participants were informed at the end of the study about the nature of each drink by the supervising investigator.

All participants were native German speakers and participation was voluntary, although students could earn credit points for a research class. Participants with medical conditions (e.g., diabetes) or food/drug consumption within the last 12 hours were excluded. All aspects of the study design were approved by the university’s internal review board (MS/BBL 191119) and the study was preregistered at the German clinical trial register (DRKS00019843).

A-priory power analyses yielded a necessary sample size of 63 participants for our estimated expected effects. Our collected sample consisted of 80 participants (27 men, 53 women). Nine data sets had to be excluded due to non-compliance with study requirements (prior food/drug consumption). The final sample included 71 participants with a mean age of 23.17 (*SD* = 6.75, range: 18–63 years) for analyzes.

### Procedure and materials

On day 1 of testing, participant information was provided and informed consent was signed. The remainder of the study protocol was identical for both sessions, starting with beverage consumption (glucose solution or water). Participants were allowed three minutes for ingestion and, to ensure adequate absorption, spent the next 20 min following a standardized protocol that preceded cognitive performance tests (learning phase of verbal recall test and anthropometric measures).

For anthropometric measurements, internationally standardized guidelines were applied [13]. Body mass index (BMI) served as an indicator for participants’ body composition, which was validated using caliper measurements of four skinfold-thicknesses (triceps, suprailiac, subscapular, and thigh).

To assess cognitive performance, a selection of established standardized tasks for different domains was used. A computerized implementation of the Corsi-Block-Tapping Task served as a measure for viso-spatial short-term memory performance [14].

A Go-No-Go Task (250 trials, 50% nogo) was used to measure inhibitory performance [15], with error rate and mean RT for correct responses as dependent variables.

The verbal recall was assessed using parallel versions of the German Rey Auditory Verbal Learning Test [16]. Depending on the time interval, retest reliability is stated to be between *r*_tt_ = 0.68 and *r*_tt_ = 0.87 [16]. The three-minute recall phase concluded the experimental part of the first session. On the second day, it was followed by a second, unannounced (long-time) recall phase of the word list from session one.

### Statistical analysis

Statistical analyzes were conducted using R [17]. Scores for differences in performance between glucose and baseline conditions were calculated for each participant and variable (CGS). For ease of interpretation, positive values of glucose-induced benefit were coded to indicate higher proficiency in the supplementation condition. Significance levels for all tests were set at *α* < 0.05 and test assumptions were met unless specified otherwise.

## Results

Descriptive statistics on physical and cognitive parameters are reported in Table s1. BMI did not differ significantly between sexes and was within the normal range (*M*_f_ = 21.67, *M*_*m*_ = 22.86; *t* = 1.68, *p* = 0.097). The relationship between skinfold-thickness and BMI (*r* = 0.48, *p* < 0.001) did not imply any added benefit for including both. For this reason and for ease of replication, the actual hypothesis tests were carried out solely based on BMI.

**Fig. 1:**
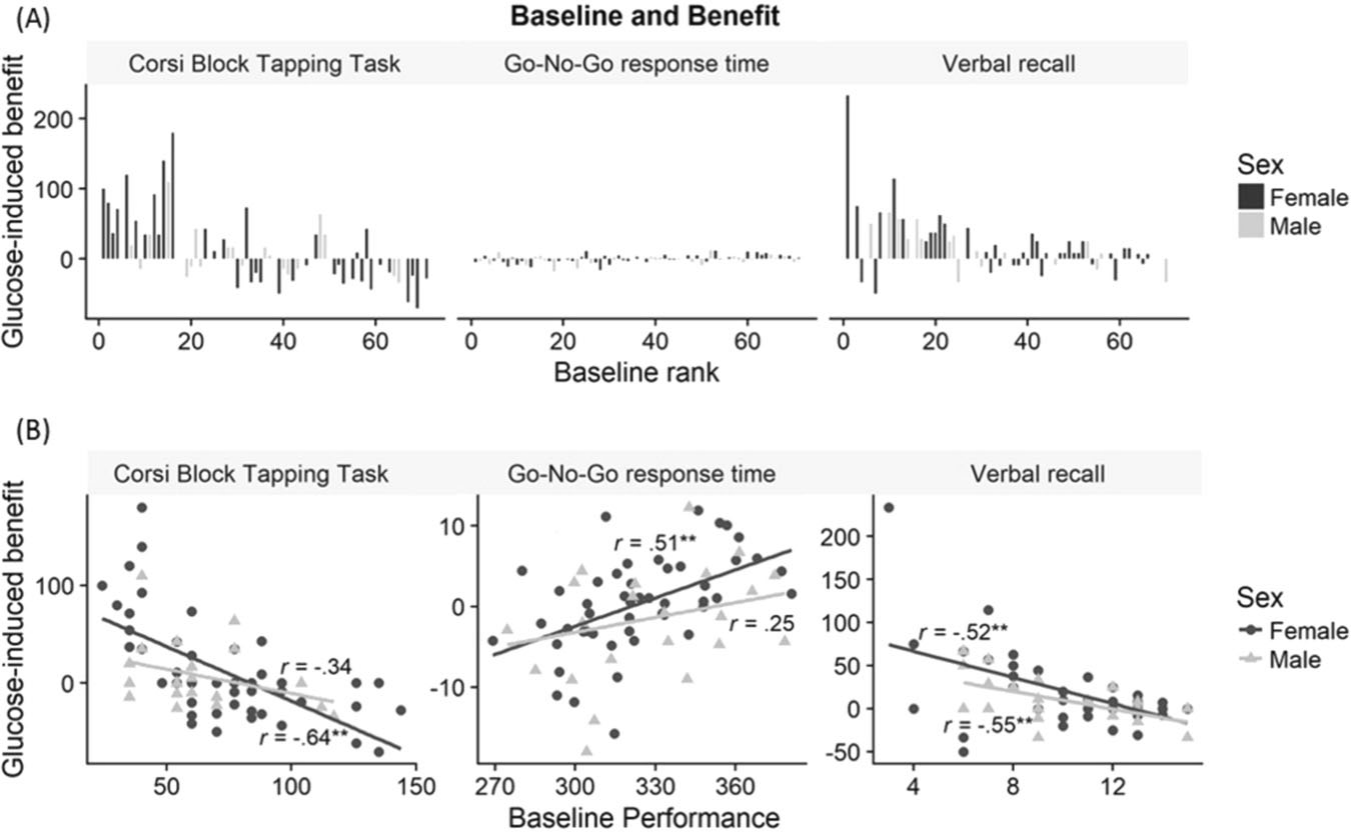
Relation between deprivation baseline performance and glucose induced benefit across tasks by sex. **A** Each bar represents one participant’s change in performance between glucose and baseline condition, expressed in percent of the baseline performance. Positive values indicate better performance (glucose-induced benefit). Baseline rank refers to the performance rank that each participant obtained in the corresponding task in the baseline condition. The lowest baseline performers are on the left while the highest baseline performers are on the right of each *x* axis. **B** Baseline performance and change in performance in response to glucose expressed in percent of performance in the baseline condition. Positive percentages indicate better performance in glucose condition (from left to right: higher score, faster response time, more words recalled). *X* axis represents absolute baseline performance (left to right: score, milliseconds, number of words recalled).

Mean performance between baseline and glucose condition did not differ substantially across tasks, however, the mean of individual glucose-induced benefit values (expressed in percent of baseline performance) reached considerable sizes (−70% to +233%, see Table s1). Especially for the Corsi-Block-Tapping Task, the mean value of all individually computed glucose-induced benefit percentages exceeds the raw difference between the mean of baseline and glucose performance. This makes sense, given that low performers experienced greater glucose-induced benefit than higher performers, which is in line with the observed pattern displayed in Fig. 1A.

Results for hypothesis tests are presented in Table 1. We found significant correlations between baseline performance and the magnitude of glucose-induced benefit for all three cognitive tasks. A one-way repeated measures ANOVA was conducted to compare the effect of task domain on individual glucose-induced benefit and indicated a significant effect of task domain, *F*(2, 140)=4.08, *p* = 0.018. Additional analyzes for sex differences revealed that female participants’ results were mirroring overall results, whereas in the male sub-sample only the verbal recall paradigm showed a significant relationship between glucose-induced benefit and baseline performance (Fig. 1B).

**Table 1:** Relationship between glucose-induced benefit and baseline performance/BMI.

	Baseline performance			BMI		
	Pearson’s *r*	*p* value	FDR	Pearson’s *r*	*p* value	FDR
Corsi-Block-Tapping Task	−0.57	<0.001***	<0.001	−0.17	0.169	0.276
Women only	−0.64	<0.001***	<0.001	−0.17	0.261	0.357
Men only	−0.34	0.112	0.202	−0.18	0.405	0.455
Go-No-Go Task	0.39	0.001**	0.002	0.13	0.290	0.357
Women only	0.51	*<*0.001***	0.001	0.26	0.076	0.152
Men only	0.25	0.245	0.357	0.04	0.856	0.857
Word list recall	−0.50	*<*0.001***	<0.001	0.02	0.857	0.857
Women only	−0.52	*<*0.001***	0.001	−0.15	0.297	0.357
Men only	−0.55	0.006**	0.015	0.49	0.017*	0.037

Go-No-Go Task performance was expressed as response time. Thus, the corresponding correlation coefficients’ algebraic signs need to be interpreted in reverse. The total sample size was 71 (48 women, 23 men). Correlations were computed one-tailed. False discovery rate (FDR) is given for each tested hypothesis. *p < 0.05, **p < 0.01, ***p < 0.001.

BMI was not significantly associated with greater glucose-induced benefit overall, but we found specific effects for sex. Specifically, for Go-No-Go response time, a relationship between women’s BMI and individual glucose-induced benefit approached significance. More importantly, in word list recall, male BMI was significantly correlated to higher glucose-induced benefit. Notably, the direction of the correlation between BMI and glucose-induced benefit in verbal recall performance was opposite between the two sexes (Fig. s1).

## Discussion

In the current study, we provided evidence for a significant relationship between individual cognitive performance in the baseline condition and individual performance gain under glucose supplementation. Furthermore, there was a significant effect of task domain on glucose-induced benefit. BMI effects occurred only for male participants with a higher performance increase under glucose supplementation in the short-term memory task.

This pattern of results provides support for our hypotheses that the benefit of glucose supplementation on cognitive performance is moderated by individual variables and performance domain. A closer look at Fig. 1 suggests a compensatory effect, especially benefiting low performing individuals, rather than a general enhancement of performance, as high performing individuals were relatively unresponsive to glucose intake.

The observation of sex-specific effects is in line with previous suggestions of a potential link between excess weight and cognitive decline in men [18] and notions about sex differences in brain metabolism [9]. This is also consistent with additional analyzes, indicating that performance in the unannounced verbal recall task was significantly negatively correlated to BMI in men but not in women. The absence of significant-general performance differences between supplementation conditions may be an indicator of the high relevance of inter-individual responsiveness, as operationalized by CGS.

On a general level, our results support the notion of a moderating role of cognitive and non-cognitive parameters in performance effects of glucose supplementation, whereas raising questions about the underlying mechanisms of the moderating parameters. Possible candidates here include factors that influence glucose homeostasis in relevant tissues, such as cerebral insulin sensitivity or accommodation to the utilization of ketone bodies [11, 19, 20].

Overall, the present study can be regarded as a proof of concept for the CGS construct, as we did find reliable effects in the assumed direction, CGS and related effects seem to be a promising research avenue. Follow-up studies with larger sample sizes should attempt to employ direct measures of blood glucose regulation or manipulate it directly, e.g., as applied in research involving intranasal insulin applications [21]. Overcoming the limitation posed by the lack of physiological measures in the presented study could also be key to investigating the role of metabolic switching in the context of CGS. In this context, the recruitment of appropriate samples could enable the investigation of the role of other glucose metabolism-related factors (e.g., age, diabetes status, activity level).

In addition, the inclusion of a second control group with a sweet-tasting placebo could help in differentiating to what extent CGS is mediated by other psychological effects, e.g., reward motivation [22]. Furthermore, expectancy effects should be regarded using appropriate questionnaires. The investigation of CGS in additional tasks could help to further disentangle the effects of task domain and difficulty. Considering the inverted u-shaped relationship between glucose uptake and performance suggested by pioneers in the field [6], CGS should also be studied in the context of different dosages.

We have presented evidence that individuals without diabetes may already show severe cognitive impairment in the absence of external glucose sources (operationalized as CGS). Behaviorally, compensating for this in everyday life, leading to a progressive dependence on frequent glucose intake in the long term, could be one pathway for increased diabetes risk. Owing to its potential behavioral influence, CGS could represent a facilitating and maintaining factor in the development of overweight. In addition, the relevance of the construct for performance optimization in non-clinical contexts, such as school nutrition, might also be explored.

Regarding the potential compensatory effects of glucose intake on low cognitive performance, we would like to encourage the investigation of CGS, its behavioral consequences, and their role in the development of—and interplay with—impaired blood glucose regulation and overweight, e.g., in a framework as proposed by Hargrave, Jones, and Davidson [23].

## Supplementary information

Figure s1

Figure Legend s1

Table s1

Table legend s1
