# Supplementary figures and images for: Cognitive glucose sensitivity—proposing a link between cognitive performance and reliance on external glucose uptake
Source: Nutr Diabetes. 2022 Mar 14;12:10. doi: 10.1038/s41387-022-00191-6 (PMC8921321; doi:10.1038/s41387-022-00191-6)

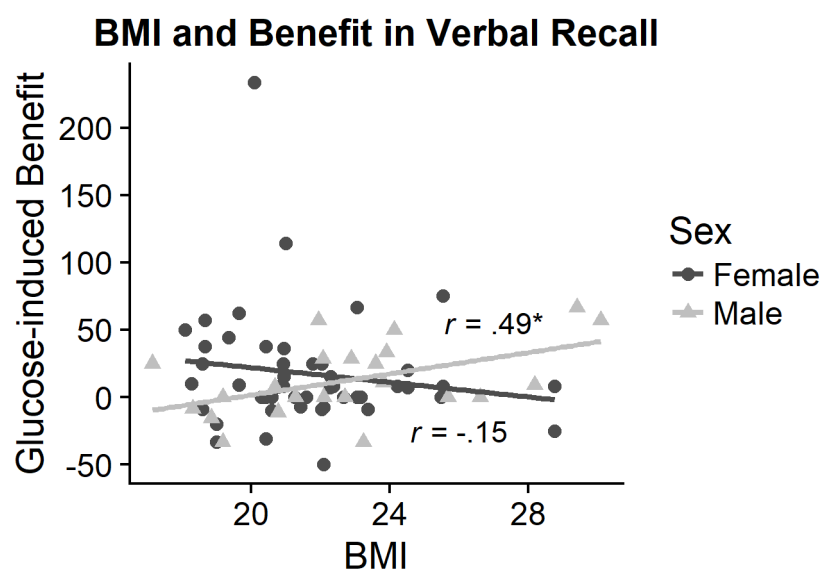

Supplement: Supplementary file 1 — Figure s1 [file 41387_2022_191_MOESM1_ESM.pdf]
