# Supplementary material for: Cognitive glucose sensitivity—proposing a link between cognitive performance and reliance on external glucose uptake
Source: Nutr Diabetes. 2022 Mar 14;12:10. doi: 10.1038/s41387-022-00191-6 (PMC8921321; doi:10.1038/s41387-022-00191-6)
Supplement: Supplementary file 2 — Figure Legend s1 [file 41387_2022_191_MOESM2_ESM.docx]

Figure s1.

BMI and change in number of correctly recalled words in response to glucose, expressed in percent of the performance in the baseline condition. Positive percentages indicate that more words were recalled correctly in the glucose condition.
