# Supplementary material for: Cognitive glucose sensitivity—proposing a link between cognitive performance and reliance on external glucose uptake
Source: Nutr Diabetes. 2022 Mar 14;12:10. doi: 10.1038/s41387-022-00191-6 (PMC8921321; doi:10.1038/s41387-022-00191-6)
Supplement: Supplementary file 4 — Table legend s1 [file 41387_2022_191_MOESM4_ESM.docx]

Table 1s. Mean, standard deviation, minimum and maximum for physical and cognitive variables of interest.
